# Metabolic Energy Correlates of Heart Rate Variability Spectral Power Associated with a 900-Calorie Challenge

**DOI:** 10.1155/2011/715361

**Published:** 2011-06-20

**Authors:** Richard M. Millis, Rachel E. Austin, Mark D. Hatcher, Vernon Bond, Kim L. Goring

**Affiliations:** ^1^Department of Physiology & Biophysics, Howard University College of Medicine, Washington, DC 20059, USA; ^2^Department of Health, Human Performance and Leisure Studies, Howard University College of Arts and Sciences, Washington, DC 20059, USA; ^3^Department of Medicine, Howard University Hospital, Washington, DC 20060, USA

## Abstract

We studied healthy males challenged with a 900 Cal test beverage and correlated EE with the raw (ms^2^) and normalized units (nu) of total power (TP), low frequency/high frequency (LF/HF) and VLF spectral power of heart rate variability (HRV). The correlations were evaluated during 20 min of normal breathing (NB, control) and 20 min of paced breathing (PB) at 12 breaths·min^−1^ (0.2 Hz). EE was not significantly correlated with any of the HRV variables before the metabolic challenge. After the challenge, EE was positively correlated with LF/HF and with VLF; VLF was also positively correlated with LF/HF during both NB and PB. These findings suggest that EE may be a correlate of LF/HF and of VLF spectral power of HRV in healthy adolescent/young adult males. The association of lower resting energy expenditure with lower amounts of VLF spectral power may occur in individuals with predilections for obese phenotypes.

## 1. Introduction

Sympathetic regulatory mechanisms are at the nexus of nutrition, metabolism, and obesity. Heart rate variability spectral power (HRVSP) measurements of respiratory sinus arrhythmia are noninvasive indicators of the autonomic influences on heart rate regulation. Advances in knowledge of obese phenotypes have been impeded by the lack of noninvasive technologies for measuring the impact of body fat on regulatory mechanisms. However, this impediment has effectively been overcome by the advent of heart rate variability (HRV) analyses for elucidating autonomic mechanisms [1] which make it possible to differentiate a wide variety of conditions with common autonomic etiologies [2–6].

Previous studies have shown correlations between increments in vagal signaling and high-frequency (HF) HRVSP during controlled (paced) breathing [7–9], and paced breathing has often been used to limit the influence of HF HRVSP on the low-frequency/high-frequency spectral power ratio (LF/HF), a commonly used index of sympathetic influences. Although the percentage of body fat may be a determinant of HRVSP measured at rest [10, 11], the influence of body fat on HRV measurements was found to be nil when performed at rest and significant only during an autonomic challenge [12]. We have demonstrated positive correlations of LF/HF with the respiratory quotient (RQ) before and after feeding [13] and negative correlations with the percentage of body fat in healthy young adult/adolescent African American males after overnight fasting and the latter only during 5 min periods of spontaneous, uncontrolled breathing and not during 5 min periods of paced breathing at 12 breaths/min, 0.2 Hz [14]. Other researchers have reported that changes in the percentage of body fat may be correlated with changes in the HRVSP attributed to the very-low-frequency (VLF) band, reported to be a measure of sympathetic thermoregulatory and metabolic energy signaling [15].

The significances of VLF to measures of metabolic energy signaling during trials of uncontrolled and paced breathing associated with different physiological states remain unclear. This may be partly because heart rate variability measurements are commonly made from 5 min electrocardiographic recordings which do not usually contain sufficient spectral power for reliable analyses [16]. Since the VLF band lies between 0.001 and 0.04 Hz, 5 min recordings, as we previously reported [13, 14], would provide 0.3–12 waves for analysis. By computation, 20 min recordings appear to provide 1.2–48 waves and sufficient power for analysis of the VLF band [17].

We designed this study to use 20 min electrocardiographic recordings for comparing the effects of normal uncontrolled breathing and paced breathing at 0.2 Hz. In addition, we determined the correlations between the sympathetic influences of HRVSP and resting energy expenditure, an important determinant of predilections for obesity. We tested the hypothesis that, in healthy resting subjects, the LF/HF index of sympathetic modulation of the heart rate and the VLF indicator of metabolic energy signaling are significantly correlated with resting energy expenditure after a significant energy metabolic challenge.

## 2. Materials and Methods

### 2.1. Study Participants and Design

This experimental protocol was approved by the Howard University Human Participants Institutional Review Board, and each subject provided informed consent. The study group consisted of ten 18–20-year-old African American male university students. Criteria for inclusion in the experiment were nonsmoking status, absence of alcohol abuse (less than two standard alcohol drinks a day), and absence of use of medication that could interfere with autonomic modulation. Each subject was studied twice, on separate days at which time they were subjected to a metabolic challenge by ingestion of a 900 Cal beverage. The data collected for each subject on separate days were averaged.

### 2.2. Uncontrolled and Paced Breathing

The subjects were instructed to breathe normally while lying recumbent at 45 degrees in a bed of the General Clinical Research Center (GCRC) at Howard University Hospital. Following 20 min of the normal uncontrolled breathing protocol, subjects were instructed to perform 20 min of paced breathing. Each subject practiced paced breathing for a period of 3–5 min and was then instructed to perform the same paced breathing maneuver for the 20 min paced breathing trial by following a visual tracking image on a computer monitor for periodic durations of inspirations and expirations set to 12 breaths*·*min^−1^ (0.2 Hz). The electrocardiogram signals were recorded using a Biopac MP100 data acquisition system (Biopac Systems, Santa Barbara, CA). The electrocardiogram electrodes were placed on the subject's chest in a standard three-lead position with recordings obtained from standard lead II.

### 2.3. Heart Rate Variability Analyses

HRV in the time domain was measured as the average standard deviation of the consecutive normal-normal electrocardiogram RR (interbeat) intervals (SDNN). Fast Fourier transform analysis of the electrocardiogram RR intervals was used to spectrally decompose heart rate variability in the frequency domain. For the frequency domain analysis, vagal respiratory modulation was represented by the area under the high-frequency power spectrum (HF: 0.15–0.4 Hz), sympathetic and vagal cardiovascular modulation by the area under the low-frequency power spectrum (LF: 0.04–0.14), and sympathovagal influences on heart rate modulation by the ratio LF/HF have been previously reported during 5 min electrocardiographic recordings [14]. In this study, we employed 20 min electrocardiographic recordings and analyzed the areas under the HF, LF, and the VLF (0.001–0.04 Hz) using specialized computational software (Nevrokard, Version 6.3, Ljubljana, Slovenia). All time and frequency domain analyses were carried out in accordance with the guidelines put forth by the *Task Force of the European Society of Cardiology and the North American Society of Pacing and Electrophysiology *[16].

### 2.4. Anthropomorphic, Cardiovascular, and Metabolic Measurements

Body weight and height were measured (Detecto scale), and these values were used to compute body mass index (BMI) as the quotient kg body weight/m^2^ height. RQ (VCO_2_/VO_2_) and resting energy expenditure were measured by indirect calorimetry using an isolated flow-directed breathing chamber (Deltatrac, Sensor Medics, Yorba Linda, CA). Percent body fat was measured by dual energy X-ray absorptiometric (DEXA) whole body scanning (Model DPX-L, Lunar Corp., Madison WI).

### 2.5. Statistical Analyses

The study design consisted of a comparison and correlation analysis of measurements of body mass index (BMI), percentage of total body fat (TBF), respiratory quotient (RQ), resting oxygen consumption (VO_2_), resting energy expenditure (EE), the average standard deviation of the consecutive normal-normal electrocardiogram RR intervals (SDNN), and the areas under the HF, LF, and VLF power spectra of HRV during 20 min trials of normal uncontrolled and paced breathing at 0.2 Hz. The significance of differences between the normal and paced breathing conditions was evaluated by analysis of variance using a multivariate general linear model with significance set at *P* < .05. A correlation analysis between the aforementioned metabolic variables and the LF/HF computed from the raw (ms^2^) and normalized units (nu) and the VLF raw power (ms^2^) was based on linear regression and Pearson's correlation coefficient during the 20 min uncontrolled and paced breathing trials with significance at *P* < .05. A statistical software package was used for the computations and analyses (SPSS, Chicago, IL).

## 3. Results

Table 1 summarizes the relevant characteristics and control measurements in the study group determined with the subjects at rest. The RQ indicates that the energy substrates of this study group consisted of a mixed diet. The LF/HF shows a predominance of vagal and a relatively small amount of sympathetic influence on heart rate regulation.

Table 2 compares the VO_2_, resting energy expenditure, SDNN, and HRVSP values for the uncontrolled breathing and paced breathing trials. The correlation coefficient for each measurement under the two different breathing conditions is also presented. Compared to normal breathing, the HRV measurements SDNN, TPms^2^, and VLFms^2^ and the metabolic variables RQ, VO_2_, and EE were increased by the 0.2 Hz paced breathing maneuver.

**Figure 1:**
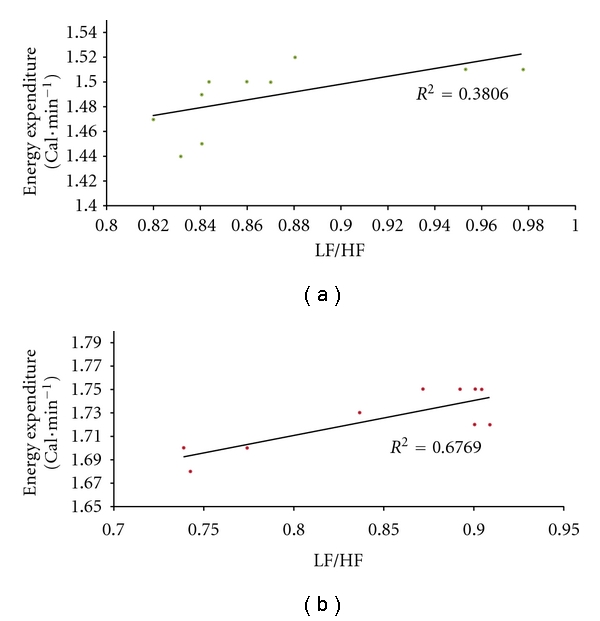
Correlation of low-frequency/high-frequency spectral power of heart rate variability with resting energy expenditure. Linear regression for low-frequency/high-frequency (LF/HF) heart rate variability spectral power computed from fast Fourier transform analysis of electrocardiogram RR intervals for ten healthy 18–20-year-old African American males during 20 min trials of uncontrolled breathing (a) and paced breathing at 0.2 Hz (b) after metabolic provocation with a 900 Cal test beverage. *R* ^2^ = .38; *P* < .05  and  .68; *P* < .01, 8 df, respectively.

**Figure 2:**
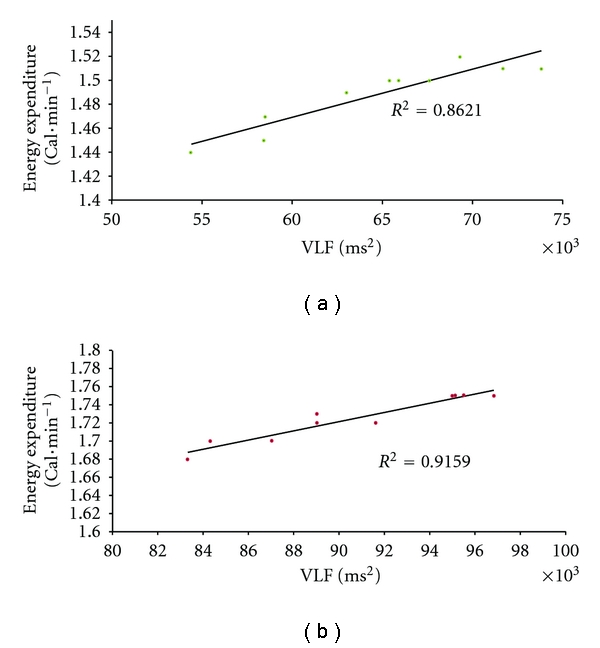
Correlation of very-low-frequency spectral power of heart rate variability with resting energy expenditure. Linear regression for raw (ms^2^) units of very-low-frequency (VLF) power computed from fast Fourier transform analysis of electrocardiogram RR intervals for ten healthy 18–20-year-old African American males during 20 min trials of uncontrolled breathing (a) and paced breathing at 0.2 Hz (b) after metabolic provocation with a 900 Cal test beverage. *R*2 = .86  and  .92; *P* < .001, 8 df, respectively.

BMI and TBF were not correlated with any of the HRVSP variables either before or after the metabolic challenge. VO_2_ and EE were not significantly correlated with any of the HRVSP variables before the metabolic challenge. Figures 1 and 2 show that, after the challenge, EE was positively correlated with LF/HF during NB (*r* = .62–0.64, *P* < .05) and during PB (*r* = .85–.86, *P* < .01) and with VLF, also during NB (*r* = .91–.92, *P* < .001) and PB (*r* = .94–.96, *P* < .001). EE was positively correlated with VO_2_ (0.96–0.98, *P* < .001), and VLF was positively correlated with LF/HF (*r* = .85–.86, *P* < .01) during both NB and PB.

## 4. Discussion

The main finding of this study is a significant positive correlation between resting energy expenditure and the VLF power of HRV during 20 min periods of uncontrolled normal breathing and 20 min periods of controlled, paced breathing at 0.2 Hz in a group of healthy adolescent/young adult African American males exhibiting a wide variation of total body fat and body mass index. This correlation was not detected under control conditions before a 900 Cal metabolic challenge. A similar positive correlation was also found for the association between resting energy expenditure and LF/HF heart rate variability spectral power, an indicator of sympathetic modulation of the heart rate. These findings suggest that low resting energy expenditure, a predictor of predilections for the development of obesity, may be associated with low values of LF/HF and of VLF heart rate variability spectral power only after a metabolic challenge such as that which might be associated with metabolizing a large meal. The VLF band appears, therefore, to be a correlate of resting energy expenditure only after a significant metabolic energy challenge.

The guidelines for standardizing heart rate variability measurements state that VLF spectral power may represent too small a proportion of the total to be worthy of analysis during relatively short intervals of electrocardiographic recordings [16]. More recent studies have suggested that the VLF band could, under appropriate conditions, be an indicator of sympathetic thermoregulatory and metabolic energy signaling [15, 18, 19]. Effective use of 20 min electrocardiographic recordings, such as those employed in the present study, provided a sufficient number of waves and power to evaluate the VLF band [17], a putative indicator of sympathetic thermoregulation and energy substrate metabolism [15]. A shift in autonomic regulation toward less sympathetic modulation is reported during the ingestion of water in normal healthy subjects [20]. The metabolism of food, however, produces a shift toward greater sympathetic modulation similar to that associated with postural changes [21–27]. Higher BMI is associated with greater sympathetic responsiveness to postural changes, higher plasma leptin levels [22], and greater lipolytic activity of adipocytes [28]. These findings suggest that inferences about the effects of high BMI, commonly associated with high percentages of body fat and tendencies for obesity, which may be based on HRV measurements, can vary with the physiological state. In the present study, we found significant increments in respiratory quotient and resting energy expenditure during the different physiological states of breathing: paced breathing at 0.2 Hz compared to uncontrolled normal breathing. Moreover, we discovered that the significant correlation between resting energy expenditure and LF/HF found during 20 min of normal breathing were not obscured during 20 min of 0.2 Hz paced breathing. These findings are in contrast to our previous report that the correlation between percentage of total body fat and LF/HF was masked by a 5 min paced breathing maneuver at 0.2 Hz and was observed only during trials of uncontrolled breathing [14]. The results of the present study did not confirm a correlation between percent body fat and LF/HF, nor between percent body fat and VLF, during 20 min periods of uncontrolled and paced breathing. 

Requirements for controlling the respiratory frequency during measurements of heart rate variability are controversial. Paced breathing is thought, by some researchers, to be necessary for controlling the respiration-related variability (respiratory sinus arrhythmia) of the electrocardiogram interbeat (RR) intervals on which heart rate variability measurements are based [7–9]. Several mechanisms have been attributed to this requirement; for example, respiratory sinus arrhythmia might be amplified by increased tidal volume [7]. We previously reported no significant difference in LF/HF during uncontrolled versus paced breathing at 0.2 Hz [14], as was the case in the present study. Respiratory frequencies controlled at 0.17 Hz, 0.25 Hz, and 0.33 Hz are reported to have no effect on LF and to modulate HF power only [8]. Increased tidal volume is reported to increase HF power [29, 30], and paced breathing at 0.2 Hz, the respiratory frequency that we used in the present study, is reported to be associated with increased tidal volumes [31]. In the present study, the subjects were lying recumbent during both the paced and uncontrolled breathing conditions, thereby, ruling out changes in sympathetic modulation associated with changes in posture. However, increased LF power has been shown to occur in association with an increased respiratory rate during conditions of mental stress [32], and, although it could have occurred in the present study because of experimental stress, differences in tidal volumes associated with paced breathing [31], or because of differences in respiratory frequency during the uncontrolled breathing trials, no differences in LF/HF, an accepted measure of sympathetic modulation, were found. Despite these unknowns, no interferences of 20 min of paced breathing at 0.2 Hz with the correlation between resting energy expenditure and LF/HF, nor with the correlation between resting energy expenditure and VLF, were detected.

## 5. Conclusion

The results of this study demonstrate that the low-frequency/high-frequency and the very-low-frequency heart rate variability spectral power were positively correlated with resting energy expenditure during 20 min periods of normal and paced breathing after a 900 Cal metabolic challenge. In view of previously reported confounding influences of 5 min of 0.2 Hz paced breathing maneuvers on the correlation between low frequency/high frequency heart rate variability spectral power and percentage of total body fat, 20 min of paced breathing at 0.2 Hz does not appear to confound the association of lower energy expenditure with smaller amounts of low frequency/high frequency and with very-low-frequency heart rate variability spectral power, as potential indicators of predilections for obesity.

##  Conflict of Interests

The authors declare no conflict of interests.

## Figures and Tables

**Table 1:** Control measurements.

Variable	Mean ± SD
Age (y)	19 **±** 1.0
Body Temperature °F	97 **±** 0.7
Body mass index	26 **±** 7
Body fat (%)	22 **±** 12
Respiratory quotient	0.9 **±** 0.2
Oxygen consumption (mL*·*min^−1^)	294 **±** 64
Energy expenditure (Cal*·*min^−1^)	1.4 **±** 0.3
Systolic blood pressure (Torr)	128 **±** 13
Diastolic blood pressure (Torr)	70 **±** 9

HRV measurements	

Standard deviation of RR intervals (ms)	89 **±** 35
Total spectral power (ms^2^ × 10^3^)	10 **±** 9
Very-low-frequency spectral power (ms^2^ × 10^3^)	2 **±** 4
Low-frequency spectral power normalized (nu)	32 **±** 17
High-frequency spectral power normalized (nu)	64 **±** 16
Low-frequency *·* high-frequency^−1^ spectral power	0.5 **±** 1.0

**Table 2:** Comparison of uncontrolled normal and paced breathing conditions.

Variable	Normal breathing	Paced breathing	*P*-value	Correlation
	mean ± SE	mean ± SE		
Respiratory quotient	0.85 **±** 0.01	1.08 **±** 0.01	*P* < .001	*r* < .55, *P* > .1
Oxygen consumption (mL*·*min^−1^)	313 **±** 18	353 **±** 17	*P* < .001	*r* = .98, *P* < .01
Energy expenditure (Cal*·*min^−1^)	1.5 **±** 0.08	1.8 **±** 0.08	*P* < .001	*r* = .98, *P* < .01
Standard deviation of RR intervals (ms)	80 **±** 6	95 **±** 10	*P* < .05	*r* = .58, *P* < .05
Total spectral power (ms^2^ *·*10^3^)	445 **±** 8	528 **±** 8	*P* < .001	*r* = .60, *P* < .05
Very-low-frequency spectral power (ms^2^ *·*10^3^)	65 **±** 2	91 **±** 2	*P* < .001	*r* = .96, *P* < .01
Low-frequency*·*high-frequency^−1^ spectral power	0.6 **±** 0.2	0.8 **±** 0.2	*P* > 0.1	*r* < .55, *P* > .1
